# Imaging of glutamate in acute carbon monoxide poisoning using chemical exchange saturation transfer

**DOI:** 10.3389/fneur.2023.1065490

**Published:** 2023-02-02

**Authors:** Hongyi Zheng, Wenbin Zheng, Hongkun Liu, Gengbiao Zhang, Weijia Li, Jiayan Zhuang, Yuelin Guo

**Affiliations:** ^1^Department of Radiology, The Second Affiliated Hospital, Medical College of Shantou University, Shantou, China; ^2^Department of Radiology, Huizhou City Center People's Hospital, Huizhou, China; ^3^Department of Radiology, Shenzhen Hospital of Integrated Traditional Chinese and Western Medicine, Shenzhen, China

**Keywords:** carbon monoxide poisoning, chemical exchange saturation transfer, CO poisoning, GluCEST, glutamate

## Abstract

**Aims:**

This study adopted the Glutamate Chemical Exchange Saturation Transfer (GluCEST) imaging technique to quantitatively analyze cranial glutamate and discussed the effectiveness of GluCEST values in identifying the pathogenesis of encephalopathy after CO poisoning.

**Methods:**

The routine MRI and functional MRI scans of two cohorts of subjects (CO group, *n* = 29; Control group, *n* = 21) were performed. Between-group comparisons were conducted for GluCEST% in regions of interest (ROI), including the basal ganglia, the thalamus, the frontal lobe, the occipital lobe, the genu of corpus callosum, the cingulate gyrus, and the cuneus. Moreover, an age-stratified subgroup analysis was devised, and a correlational analysis was performed for GluCEST% in each ROI, including the time in coma, Simple Mini-Mental State Examination Scale (MMSE) score, Hamilton Anxiety Scale score, and blood COHb%.

**Results:**

As compared to the healthy control, the CO group led to significantly increasing GluCEST% in the basal ganglia, the occipital lobe, the genu of the corpus callosum, the cingulate gyrus, and the cuneus (*p* < 0.05). In the subgroup analysis for age, adult patients had higher GluCEST% in the basal ganglia, the thalamus, the occipital lobe, the cingulate gyrus, and the cuneus compared to healthy adults (*p* < 0.05). In addition, the correlational analysis of CO-poisoned patients revealed a statistical association between the GluCEST% and the MMSE in the thalamus and the genu of the corpus callosum.

**Conclusion:**

The GluCEST technique is superior to routine MRI in that it can identify the cerebral biochemical changes sooner after acute CO poisoning, which is significant for our understanding of the role of neurotransmitters in the pathological basis of this disease. Brain injury caused by CO poisoning may be different in adults and children.

## 1. Introduction

Carbon monoxide (CO), which is tasteless, colorless, and odorless, is absorbed into the human body across the lining of the lungs (1). It is the main cause of death related to poisoning in multiple countries, and more importantly, it may lead to fetal poisoning in over half of the global population (2). People surviving CO poisoning still display long-term neurocognitive sequelae associated with cerebral injury and may present with symptoms including memory decline, cognitive impairment, depression, anxiety, and/or vestibular and motor dysfunctions (3). Given the affinity to CO is 250 times higher than oxygen (4), hemoglobin (Hb) in blood circulation can bind with CO to form carbonylhemoglobin (COHb). In that way, the Hb fails to carry more oxygen and the degradation of oxygenated hemoglobin will be blocked, leading to severe tissue hypoxia. The decreasing oxygen levels and mitochondrial oxidative phosphorylation will cause ischemic and hypoxic cerebral injuries that finally result in cognitive impairment (5). Ischemic brain injury might be a result of excitotoxicity, acidosis, ion imbalance, depolarization, oxidative stress, nitrosative stress, inflammation, and cell apoptosis (6). Excess CO activates the platelets and amplifies the inflammatory effects by triggering neutrophil activation, adhesion, and degranulation. These inflammatory effects are ongoing long after the initial CO poisoning and may dominate the clinical expression (7, 8). Adenosine triphosphate is the direct energy source of the organism. When its content decreases, intracellular protease and lipase will be activated accordingly to induce mitochondrial membrane depolarization, cell death, and release of neurotransmitters (especially, glutamate) (6, 9). Glutamate is a core player in excitatory neurotransmission. In the cases of impaired homeostasis, it will induce the production of neurotoxins or excitotoxins and activate the pathways of neuron death (10). In the presence of acute ischemic or toxic injury and chronic neurodegeneration, the glutamate receptor will be activated excessively, which is the key to the generation of excitotoxicity and incidence of cell death (11). Previous studies have reported that the most common manifestations of MR after CO poisoning are high signals in the bilateral globus pallidus and the white matter region of the brain (12, 13). Conventional MRI could show the involvement of globus pallidus with CO exposure as it is at risk for damage due to the hypotension-hypoxia processes because of poor collateral blood flow or from CO binding to heme iron in the globus pallidus, where the highest concentration of iron in the brain is located (1). The abnormal finding detected on MR imaging in the white matter is more responsible for the chronic symptoms than the gray matter (14). The centrum semiovale and periventricular white matter are the most common regions of white matter to be affected after CO poisoning (15, 16). The white matter damage can be observed in various other regions rather than the centrum semiovale and the periventricular white matter, such as the temporal lobe, the occipital lobe, the parietal lobe, and the corpus callosum (12, 17–19).

Chemical exchange saturation transfer (CEST) is a novel MRI technique. It uses frequency-selective radiofrequency pulses to ensure magnetization saturation from some exchangeable protons in solutes and characterize the microenvironment of the tested solution by metabolite concentrations, temperature, and pH values by calculating the proportional change in water signals in the bulk water pool (20–22). Comparatively, CEST is superior to magnetic resonance spectroscopy with higher specificity and spatial resolution (23). Given the advantages, CEST is widely adopted in the examination of multiple molecules, such as glucose with GlucoCEST (24), adenosine triphosphatase in polypeptides, tissue mobile proteins with amide proton transfer (25), and glutamate with GluCEST (26, 27). The asymmetric magnetization transfer ratio (MTRasym) can be calculated to represent the signal intensity on CEST, majorly by the difference in the values of the bilateral resonance frequency of the solvent water protons (ΔMTRasym). It is reported that GluCEST has high signal intensity at ΔMTRasym (3 ppm) (23) and ΔMTRasym. Therefore, quantitative calculation of the changes in glutamate concentration can be performed. Currently, there were some studies on magnetic resonance imaging (MRI) for cerebral metabolites after acute CO poisoning (28, 29), while less is devoted to the cranial glutamate changes (30, 31). Glutamate chemical exchange saturation transfer (GluCEST) imaging is a non-invasive quantitative technique and its application values in patients with acute CO poisoning are not yet reported.

Glutamate is a type of excitatory neurotransmitter most abundant in the brain and involved in a variety of physiological functions in the nervous system (32, 33). It is known that glutamate is important in cerebral injury after CO poisoning. In an animal experiment, for instance, increases in glutamate release and hydroxyl free radical production were observed during and after the hypoxia is induced by CO absorption in rats, which were believed to be the cause of ischemic cerebral injuries (30). Other studies revealed that glutamate could aggravate cellular dysfunction and cell apoptosis by activating the N-methyl-D-aspartate receptor (NMDAR) (6), and the NMDAR antagonist could alleviate the CO-induced neurodegeneration (34). We assumed that GluCEST may be effective as a precise diagnostic approach for encephalopathy during the acute phase after CO poisoning.

As a non-invasive and quantitative imaging technique, GluCEST was previously applied to multiple diseases of the nervous system (35–37). Currently, there is no report on its application in encephalopathy after CO exposure. Herein, we used a 3.0-Tesla MR scanner to assess the value of the GluCEST technique in diagnosing cerebral injuries after CO poisoning and in understanding the pathogenesis.

## 2. Methods

### 2.1. Subjects

Overall, there are 29 patients with acute CO poisoning included and categorized into the CO group. These patients were treated for acute CO poisoning in our hospital at different time points from December 2020 to March 2021. There were 12 men and 17 women, aged between 6 and 48 years (average, 19.4 ± 12.5 years). The inclusion criteria (38) included (1) a history of exposure to high concentrations of CO; (2) symptoms and signs of acute central nervous system injuries; (3) timely blood COHb content conforming to the national diagnostic criteria. For MRI, the time interval to be exposed to high concentrations of CO was required to be smaller than 3 days. Hyperbaric oxygen and correction for electrolyte disturbance treatment were performed upon admission. Adult patients with CO poisoning were treated with hormone therapy for 3 days after admission to prevent immune inflammatory responses. The patients received 1 h hyperbaric oxygen therapy (0.2 MPa) 1–2 h after admission and then received hyperbaric oxygen therapy (0.2 MPa) once a day, 1 H each time. The duration was generally 7 days. After treatment, the patient still showed clinical symptoms and cognitive decline. Although the clinical symptoms recovered, the patient did not meet the criteria for recovery. In the meantime, each patient was subjected to a Mini-Mental State Examination (MMSE), and the period in coma and COHb concentration were recorded (Supplementary Table 1). At 1 month follow-up, onsite and telephone interviews were conducted and the Hamilton Anxiety Scale (HAMA) score was obtained (Supplementary Table 1). Healthy volunteers matched for gender, age, and educational level were recruited from our hospital as the control group (control group, *n* = 21), including 9 men and 12 women aged between 8 and 30 years (average, 20.7 ± 7.4 years). In this population, there was no history of cerebral injury, psychiatric disorder, alcohol abuse/substance dependence, or diseases of the nervous system (including stroke, seizure, and somatic disease). Table 1 shows the detailed demographic and clinical information. All patients gave written informed consent. The project was approved by the Ethics Committee of The Second Affiliated Hospital of Shantou University Medical College.

**Table 1:** Participant clinical characteristics.

	**Patient**	**Control**
Gender (male/female)	12/17	9/12
Age (years)	19.4 ± 12.5	20.7 ± 7.4
MMSE (point)	16 (7.5)^*^	25.82 (4.18)^*^
Coma time (minute)	36 (30)	–
COHb (%)	33.1 (17.25)	–
HAMA (point)	2 (1.75)	2 (1)

MMSE, Simple Mini-Mental State Examination Scale; HAMA, Hamilton Anxiety Scale;

* P < 0.05, which were obtained by a two-sample t-test for age, MMSE, and the chi-square test for gender difference.

### 2.2. MRI imaging

The structural MRI and CEST MRI data were collected using a 3.0 Tesla MRI scanner (Sigma; GE Healthcare, Milwaukee, WI, USA), using an 8-channel phased-array head coil. The sponge padding was used to limit head motion. The T2-weighted images (T2WI) [repetition time (TR) = 4,600 ms, echo time (TE) = 120 ms, 20 slices, and acquisition time: 1 min 35 s], the T2WI fluid-attenuated inversion recovery images (TR = 8,600 ms, TE = 155 ms, inv. time = 2,100 ms, 20 slices, and acquisition time: 1 min 53 s), and diffusion-weighted images (TR = 5,200 ms; TE = minimum; *b* = 1,000, 20 slices, and acquisition time: 42 s) were obtained to acquire the information of the brains of all subjects.

**Figure 1:**
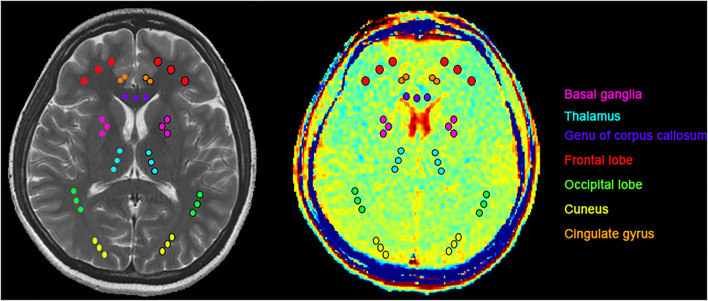
ROIs for T2WI and GluCEST: basal ganglia (pink), thalamus (light blue), the genu of the corpus callosum (purple), frontal lobe (red), occipital lobe (green), cuneus (yellow), cingulate gyrus (orange). Except for the basal ganglia region and the thalamus, the ROI was placed in the gray matter region, and for other brain regions, the ROI was placed in the white matter region.

The CEST scan was based on an MT-prepared gradient echo MRI sequence with the following settings: TR = 50 ms, TE = 3.1 ms, field of view = 240 × 240 mm^2^, matrix = 128 × 128, 1 slice, slice thickness = 5 mm, and bandwidth = 15.63 kHz. The MT saturation pulse was a Fermi pulse with a 20 ms width and a B1 of 1.95 μT (39). The CEST imaging was performed on the brain slice shown in Figure 1. The regions of interest (ROI) consisted of seven bilateral standard regions in all patients with acute CO poisoning and healthy controls which are basal ganglia, thalamus, the genu of the corpus callosum, the frontal lobe, the occipital lobe, the cuneus, and the cingulate gyrus (Figure 1). Except for the basal ganglia region and the thalamus, the ROI was placed in the gray matter region, and for other brain regions, the ROI was placed in the white matter region of the genu of the corpus callosum, the frontal lobe, the occipital lobe, the cuneus, and the cingulate gyrus. The images were interpreted independently by two experienced radiologists who were blinded to the neurological manifestations and the results of the analyses.

### 2.3. Data processing

All CEST image processing was performed using software routines written in Matlab 7 (Mathworks, Natick, MA, USA). The acquired images were corrected for B0 inhomogeneity using a water saturation shift referencing map. The corresponding correction algorithm referred to a previous detailed discussion (40). Then, the GluCEST contrast map was generated using the following equation (41).


(1) $$\begin{array}{l} G\text{lu}CEST=\frac{S\left(-3ppm\right)-S\left(+3ppm\right)}{S0} \end{array}$$

where S (−3 ppm) and S (+3 ppm) are the images obtained at −3 ppm and +3 ppm, respectively. The Z-spectra were obtained from the normalized CEST images. The MTRasym maps were computed using the equation (41).


(2) $$\begin{array}{l} MTR\text{asy}m=\frac{Ssat\left(-\Delta \omega \right)-Ssat\left(+\Delta \omega \right)}{S0} \end{array}$$

### 2.4. Statistical analysis

Data processing and analyses were completed with SPSS 20.0 (SPSS 20.0, IBM, Armonk, NY). Comparative results, MMES score, time in coma, COHb concentration, and postoperative HAMA score were represented by mean (interquartile range). Age (continuous variable) was displayed in mean ± standard deviation. The categorical variables are shown in integers. The normal distribution of continuous parameters was determined by the Shapiro–Wilk test, followed by the Kruskal–Wallis *H*-test for data comparison.

The average GluCEST% in the bilateral ROI in the two groups was measured by GluCEST, and the data that did not conform to normal distribution were tested by the Mann–Whitney *U*-test. A comparison of the age and educational levels were completed by the two-sample *t*-test and that for gender using the chi-square test. A Spearman correlational analysis was performed to calculate the association of GluCEST% with the time in coma, MMSE score, COHb concentration, and postoperative HAMA score. A *p* < 0.05 demonstrates a statistically significant difference.

## 3. Results

### 3.1. Clinical data

The reason for CO poisoning in coma patients was the long bathing time in an enclosed bathroom using the gas stove. There were no statistically significant differences between the patients and the healthy volunteers in terms of gender (χ^2^ = 0.011 and *P* = 0.917) and age (*P* = 0.65, two-sample *t*-test). The MMSE score was lower in patients with CO poisoning (*P* < 0.001, two-sample *t*-test). In the acute phase, patients presented with symptoms, including coma, headache, dizziness, fatigue, and memory decline after a lucid interval. Upon follow-up 1 month after the discharge, the HAMA scores between the two groups were not remarkably different. There was no evident sequela, except for one who still had dizziness and memory decline. The demographic and clinical information of the subjects of the study are shown in Table 1. There were no statistical differences between the subgroups of age regarding COHb concentration, the time in coma, and the HAMA score upon follow-up.

### 3.2. MRI manifestations

**Figure 2:**
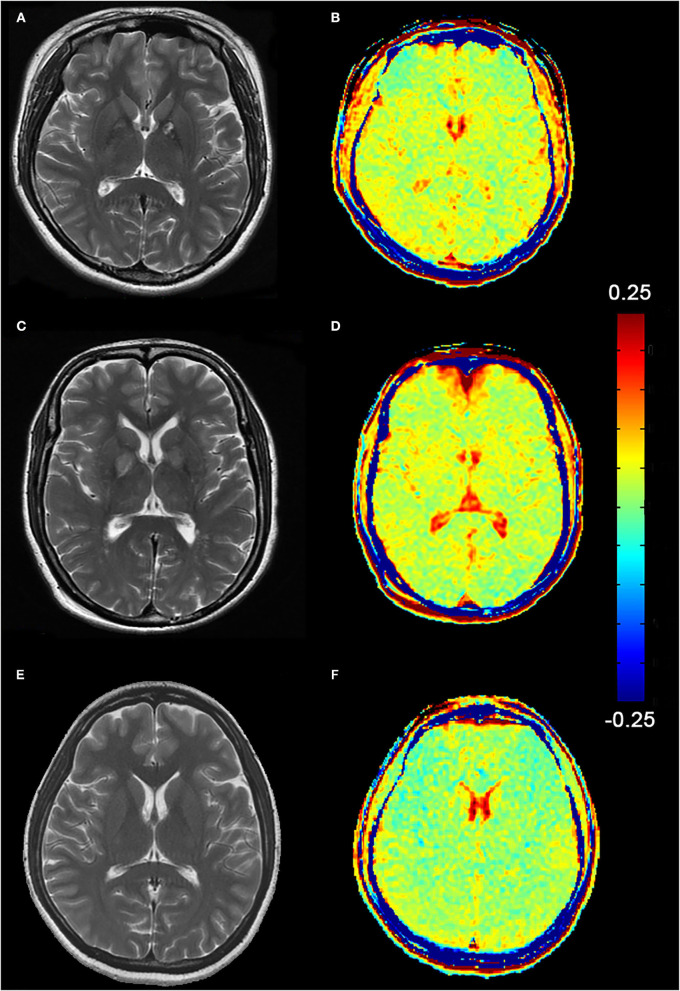
**(A, C)** Abnormal T2WI images of two female patients (Patient A, 24-year-old and Patient C, 47-year-old), mainly manifested in the bilateral globus pallidus. **(B, D)** Higher signals on GluCEST images present in the globus pallidus and the cerebral white matter region. **(E, F)** T2WI and GluCEST images of a healthy adult (female, 24-year-old).

**Figure 3:**
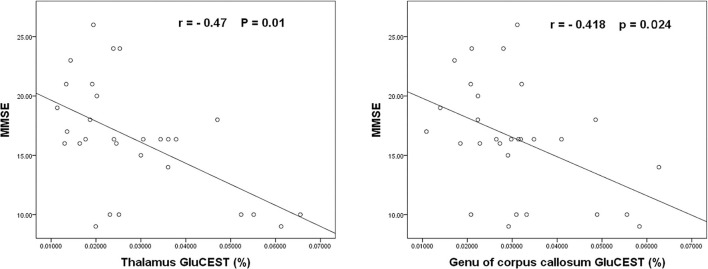
The negative association of the GluCEST% in the thalamus and the genu of the corpus callosum with the MMSE scores of patients with CO poisoning.

In routine MRI, abnormal images were shown only in two adult patients, majorly bilateral globus pallidus on T2WI (Figure 2) and signal enhancement on T2flair and DWI. One of them also showed abnormal images in the white matter of the right occipital lobe. No abnormality was observed in the other patients. Higher GluCEST imaging parameters were generally observed in patients with CO poisoning vs. healthy volunteers (Figure 2). In terms of the GluCEST% in each ROI, it was higher in patients in the basal ganglia (*Z* = 3.253), the occipital lobe (*Z* = 4.142), the genu of the corpus callosum (*Z* = 2.323), the cingulate gyrus (*Z* = 1.96), and the cuneus (*Z* = 2.849) as compared to the healthy controls (P < 0.05, Tables 2, 3). In the subgroup analysis for age, higher GluCEST% was observed in the basal ganglia (*Z* = 3.956), the thalamus (*Z* = 2.11), the occipital lobe (*Z* = 3.429), the cingulate gyrus (*Z* = 2.506), and the cuneus (*Z* = 3.165) in adult patients, when compared to the healthy adult volunteers (*P* < 0.05, Tables 2, 3). In children, higher GluCEST% was also observed in multiple ROIs in patients, while the differences among those in healthy children were not statistically significant. Further correlational analysis revealed that the increasing GluCEST% in the thalamus and the genu of the corpus callosum in patients with CO poisoning was remarkably associated with the MMSE score (Figure 3). There was no statistical association between the GluCEST% in each ROI and the time in coma, COHb concentration, and postoperative HAMA score.

**Table 2:** GluCEST in each region of interest.

	**Basal ganglia**	**Thalamus**	**Frontal lobe**	**Occipital lobe**	**Genu of the corpus callosum**	**Cingulate gyrus**	**Cuneus**
CO poisoning	0.0352 (0.0263)	0.0242 (0.0173)	0.0294 (0.0111)	0.0444 (0.0115)	0.0295 (0.0121)	0.0331 (0.0377)	0.0338 (0.0372)
Adult CO poisoning	0.0361 (0.0557)	0.0215 (0.0349)	0.0298 (0.0167)	0.0436 (0.0139)	0.0251 (0.0322)	0.0331 (0.0227)	0.0294 (0.0261)
Children CO poisoning	0.0352 (0.0248)	0.0248 (0.017)	0.0289 (0.0095)	0.0443 (0.0132)	0.0303 (0.0081)	0.0341 (0.0759)	0.037 (0.0383)
Control	0.0200 (0.0179)	0.0173 (0.0123)	0.0276 (0.0127)	0.0244 (0.0134)	0.0224 (0.0108)	0.0235 (0.0299)	0.0171 (0.0212)
Adult control	0.0159 (0.005)	0.0159 (0.0043)	0.0231 (0.0129)	0.0188 (0.0095)	0.0193 (0.0081)	0.0209 (0.0176)	0.0171 (0.0077)
Child control	0.0433 (0.0478)	0.0272 (0.021)	0.0284 (0.0187)	0.0287 (0.0219)	0.0287 (0.0213)	0.0433 (0.0945)	0.0387 (0.0474)

GluCEST value of each group and ROI was expressed in median (interquartile range).

**Table 3:** Two-sample Mann–Whitney *U-*test in each group.

	**Basal ganglia**	**Thalamus**	**Frontal lobe**	**Occipital lobe**	**Genu of the corpus callosum**	**Cingulate gyrus**	**Cuneus**
CO poisoning vs. healthy control	3.253^**^	1.939	1.596	4.142^**^	2.323^*^	1.96^*^	2.849^**^
Adult patient vs. adult control	3.956^**^	2.11^*^	1.846	3.429^**^	1.714	2.506^*^	3.165^**^
Child patient vs. child control	0.000	0.36	0.206	1.594	0.36	0.206	0.257

Mann–Whitney U-test was performed for comparisons between the CO and healthy control groups and between the subgroups.

* P < 0.05,

** P < 0.01.

### 3.3. Discussion

CEST is a relatively new contrast mechanism of MRI. It uses the specific MR frequency (chemical shift) at the molecular level and the standard MRI technique to generate images of good spatial resolution. Some CEST techniques, such as GluCEST, can indirectly examine molecules by combining the specificity and the spatial resolution of MRI (42, 43). GluCEST is effective in measuring the glutamate content in the brain, which is generally higher than the concentration of other metabolites in the brain (42). In the current study, changes in cranial glutamate levels were examined in the acute phase after CO poisoning to study the potential effect on cranial neurotransmitters, which was not previously reported.

In a previous animal experiment, hypoxia was induced in rats by CO inhalation, and instantly, the release of glutamate and the production of hydroxyl free radicals increased in the cerebral cortex and the hippocampus, which were considered to be the cause of ischemic brain injury after CO poisoning (30). Other studies revealed that glutamate could aggravate cellular dysfunction and cell apoptosis by activating NMDAR (6). Here, we found that patients with CO poisoning had remarkably increasing GluCEST% in the basal ganglia, the occipital lobe, the corpus callosum, the cingulate gyrus, and the cuneus, indicative of more cranial excitatory neurotransmitters and a tendency of accumulation in the above brain regions. Autopsy findings suggested that the major pathological changes after CO poisoning were the necrosis of globus pallidus and demyelination in the cerebral white matter area (44–46), which were mainly attributed to cerebral ischemia and edema. Given the hyperactivity and sufficient blood supply of neurons in the gray matter regions, such as the deep brain nucleus, the gray matter is more susceptible to poisoning-induced brain ischemia than the white matter. In the subacute phase, DWI manifestations are a hypointense signal and a high ADC value in the globus pallidus and a hyperintense signal and a low ADC value in the cerebral white matter (47). This suggests that the injury in cerebral white matter is a result of both ischemic globus pallidus and progressive demyelination changes (48). In the present study, only two patients exhibited abnormal images in MRI and no abnormality was noted in the other patients. It is tempting to assume that the GluCEST technique can show early changes in cranial neurotransmitters after hypoxia induced by CO exposure, and the changes can involve the deep brain nucleus and white matter simultaneously. It is reported that the bilateral basal ganglia, especially the globus pallidus, is the most susceptible region to CO poisoning (45). Controversially, some researchers report that the changes in the striatum after CO poisoning were independent of glutamate receptor activation induced by the increasing extracellular glutamate content (49). Tambasco et al. (10) revealed that CO poisoning leads to impaired glutamate homeostasis and significantly affects neurons by producing neurotoxins or excitotoxins and activating the pathways of neuronal death. In this context, the specific mechanism of the action of glutamate elevation in the basal ganglia requires further research. Herein, we also found that the GluCEST% was abnormally increased in the bilateral occipital lobe areas, except for the common bilateral basal ganglia. The occipital lobe is the visual center and when an injury occurs, visual impairment will be developed. A previous study found that COHb >30% could result in visually evoked abnormal potentials and that CO poisoning came with a series of clinical symptoms, including ocular discomfort, blurred vision, and visual field defects, which are not entirely consistent with the findings in ophthalmic examinations (50). This is in line with our findings. In the injured areas of the cerebral white matter, the semioval center and the white matter next to the lateral ventricle are the most susceptible, and the corpus callosum can also be affected. Research revealed that corpus callosum could present with generalized atrophy after CO poisoning (18), which was once reported to be associated with neuropsychological presentations in other diseases (51). Our results showed that the GluCEST% in the genu of the corpus callosum was statistically different between the patients and healthy volunteers. In addition, the GluCEST% in the thalamus and the genu of the corpus callosum were negatively associated with the MMSE score, which was weak. This infers that the changes in glutamate levels in the genu of corpus callosum may indicate the disease condition in patients to some extent. The thalamus is key to cognitive tasks, consciousness, and awakening. Impairment of the thalamus and the connections may cause damage to a wide range of neurological functions, which might be clinically translated into significant cognitive, physical, and psychic disorders (52). We also noted that there was no correlation with the MMSE score in the other ROIs. The possible explanation might be that the MMSE score mainly targets the directional and verbal functions. There is a certain rate of false negative score when applied for moderate to critical cognitive disorders, and it is readily affected by the speech and educational levels of the patients.

The age range of patients included in this study was large and ranged from childhood to middle age. However, there are no unified reports on the changes in craniocerebral metabolites at different stages of craniocerebral development, especially glutamate. Although the patients included in this study have a large age span, in addition to the measurement analysis of all patients with CO poisoning, adults and children are also divided into different subgroups for analysis, and different results are obtained. In the subgroup analysis for age, GluCEST% in both the adult and child patients increased when compared to the corresponding healthy control. Notably, there was no statistical significance in the difference in children. We reasoned that the varying collateral circulation compensation mechanism between adults and children (53) may be used to explain the less damage to the basal ganglia region in the early acute phase after CO exposure in children. Some previous literature suggested that patients with CO poisoning could be divided into mild, moderate, and severe according to the blood COHb value, symptoms, and coma state (54, 55). However, this classification was not well-suited to the patients we included in the study. First of all, according to clinical symptoms, the conditions of our patients were relatively consistent, whose state of consciousness was manifested as mild to moderate coma, and they recovered after rescue without obvious complications. Previous literature state that the degree of poisoning in patients can be determined according to the standard of blood COHb concentration. However, clinical symptoms of acute CO poisoning and their severity do not always correlate with the concentrations of CO-Hb on admission (8, 56–58). The COHb level in the clinical diagnosis of CO poisoning is not significant (58). Herein, we also found that there was no statistical correlation between the GluCEST% and COHb concentration after CO poisoning.

At follow-up, patients made a good recovery of mental status according to postoperative HAMA scores. There was no evidence of significant sequela, and the time in coma was independent of the GluCEST values. This indicates that timely treatment was achieved in patients after coma was induced by CO poisoning and the early increase in GluCEST values might not cause irreversible damage to the brain. There are two possible explanations. First, the time in coma lacks subject assessment. Here the value was obtained mainly by the bath time, which is changeable and objective possibly affecting the data correlations. Second, patients included in this study had different degrees of CO exposure and are not only from the most critically affected populations. Only in the cases of severe CO exposure, the patients might be more likely to develop higher GluCEST values and severe clinical symptoms. More significant associations might be present, while further validation is in demand.

## 4. Limitations

The strength of this study is that it evaluates the value of the GluCEST technique as a new method in the diagnosis and understanding of the pathogenesis of brain injury after CO poisoning. However, there are still some limitations in this study. First, due to the obvious seasonal recruitment of patients with CO poisoning in our area, the number of cases collected in this study is relatively small, which may affect the experimental results. In the future, we will increase the sample size to consolidate our experimental results. Second, the grouping used in this study was based on age. According to the clinical symptoms of the patients and the different periods of CO poisoning and prognosis, patients with CO poisoning can also be divided into other subgroups. Finally, the participants in this study included subjects with a large age span. Such samples are vastly different in terms of brain development and life trajectories and could affect the results. In the future, we will increase the sample size to further explore the ability of the GluCEST technique as a new method of diagnosis and evaluation of the prognosis of CO poisoning in different subgroup analyses.

## 5. Conclusion

The GluCEST technique can provide a view of the glutamate concentration in the early phase after CO poisoning. It is superior to routine MRI as it can identify cerebral biochemical changes earlier in the acute phase after CO exposure, which is significant for our understanding of the role of neurotransmitters in the pathological basis of this disease. Cerebral injuries after CO poisoning might vary among adults and children. The early glutamate concentrations of the thalamus and the corpus callosum may be of importance in the assessment of the degree of cerebral injury after CO poisoning. The GluCEST imaging technique provides a new way to understand the pathophysiological mechanisms of CO poisoning.

## Data availability statement

The original contributions presented in the study are included in the article/Supplementary material, further inquiries can be directed to the corresponding author.

## Ethics statement

The studies involving human participants were reviewed and approved by Ethics Committee of the Second Affiliated Hospital of Shantou University Medical College. Written informed consent to participate in this study was provided by the participants' legal guardian/next of kin.

## Author contributions

HZ and WZ designed the research. HZ wrote the article. WZ contributed to the manuscript revision and reading and approved the submitted version. JZ, WL, and GZ completed the data collation. YG, GZ, and WL performed the research and analyzed the data. All authors contributed to the article and approved the submitted version.
