# Supplementary material for: Imaging of glutamate in acute carbon monoxide poisoning using chemical exchange saturation transfer
Source: Front Neurol. 2023 Feb 2;14:1065490. doi: 10.3389/fneur.2023.1065490 (PMC9932694; doi:10.3389/fneur.2023.1065490)
Supplement: Supplementary file 1 [file Table_1.DOCX]

**Supplement Table 1. Details of all CO poisoning patients**

| CO patient | Age  (years) | gender | COHb% | Coma time(min) | MMSE | HAMA | MRI scan time after CO poisoning(days) | MRI finding |
| --- | --- | --- | --- | --- | --- | --- | --- | --- |
| SUB001 | 31 | Male | 45.3 | 15 | 18 | 1-headache | 2 | Normal |
| SUB002 | 28 | Female | 30.2 | 60 | 16 | 2-dizziness | 1 | Normal |
| SUB003 | 26 | Female | 22.6 | 45 | 23 | 1-headache,1-scare | 2 | Normal |
| SUB004 | 23 | Female | 3.9 | 60 | 26 | 1-headache | 2 | Normal |
| SUB005 | 26 | Female | 40 | 30 | 15 | 2-chest distress | 1 | Normal |
| SUB006 | 48 | Male | 55.4 | 120 | 10 | 1-headache,1-constipation | 1 | Normal |
| SUB007 | 44 | Female | 27.7 | 80 | 10 | 2-poor memory | 1 | abnormal bilateral globus pallidus signal |
| SUB008 | 38 | Female | 23.7 | 20 | 17 | 1-dizziness | 1 | Normal |
| SUB009 | 47 | Female | 7 | 50 | 24 | 1-muscle pain | 2 | abnormal bilateral globus pallidus signal |
| SUB010 | 30 | Female | 52.8 | 30 | 18 | 2-nervous | 1 | Normal |
| SUB011 | 11 | Male | 24.3 | 60 | 15 | 2-dizziness,1-scare | 1 | Normal |
| SUB012 | 11 | Male | 41.2 | 80 | 17 | 1-headache | 1 | Normal |
| SUB013 | 11 | Female | 50 | 30 | 15 | 2-nightmare | 1 | Normal |
| SUB014 | 10 | Male | 30.6 | 120 | 18 | 3-dizziness | 1 | Normal |
| SUB015 | 13 | Female | 51.9 | 70 | 19 | 1-diarrhea | 3 | Normal |
| SUB016 | 7 | Male | 33.1 | 50 | 10 | 2-headache,1-scare | 1 | Normal |
| SUB017 | 12 | Female | 23.4 | 30 | 17 | 2-dizziness,1-dry mouth | 1 | Normal |
| SUB018 | 13 | Male | 28.6 | 90 | 24 | 2-nervous | 2 | Normal |
| SUB019 | 10 | Female | 39 | 36 | 16 | 2-dizziness | 1 | Normal |
| SUB020 | 6 | Male | 32.3 | 20 | 16 | 1-headache,1-muscle pain | 2 | Normal |
| SUB021 | 12 | Male | 41.5 | 30 | 15 | 3-dizziness | 1 | Normal |
| SUB022 | 11 | Female | 0.7 | 20 | 21 | 2-dizziness | 2 | Normal |
| SUB023 | 12 | Female | 27.7 | 40 | 20 | 2-scare | 2 | Normal |
| SUB024 | 10 | Male | 33.1 | 50 | 10 | 1-dizziness,1-nervous | 1 | Normal |
| SUB025 | 11 | Female | 34.1 | 20 | 21 | 1-headache | 2 | Normal |
| SUB026 | 13 | Male | 27.8 | 30 | 10 | 3-insomnia | 1 | Normal |
| SUB027 | 24 | Female | 60 | 30 | 15 | 3-dizziness | 1 | Normal |
| SUB028 | 8 | Female | 33.7 | 15 | 14 | 3-dizziness | 1 | Normal |
| SUB029 | 16 | Male | 45 | 30 | 16 | 1-palpitation | 1 | Normal |
